# An Improved Micropropagation Protocol by *Ex Vitro* Rooting of *Passiflora edulis* Sims. f. *flavicarpa* Deg. through Nodal Segment Culture

**DOI:** 10.1155/2015/578676

**Published:** 2015-07-26

**Authors:** Mahipal S. Shekhawat, M. Manokari, C. P. Ravindran

**Affiliations:** Biotechnology Laboratory, Department of Plant Science, M.G.G.A.C., Mahe, Pondicherry 673311, India

## Abstract

A procedure for rapid clonal propagation of* Passiflora edulis* Sims. f.* flavicarpa* Deg. (Passifloraceae) has been developed in this study. Nodal explants were sterilized with 0.1% HgCl_2_ and inoculated on Murashige and Skoog (MS) basal medium. The addition of 2.0 mgL^−1^ 6-benzylaminopurine (BAP) to MS medium caused an extensive proliferation of multiple shoots (8.21 ± 1.13) primordial from the nodal meristems. Subculturing of these multiple shoots on the MS medium augmented with 1.0 mgL^−1^ of each BAP and Kinetin (Kin) was successful for the multiplication of the shoots* in vitro* with maximum numbers of shoots (25.73 ± 0.06) within four weeks of incubation. Shoots were rooted best (7.13 ± 0.56 roots/shoots) on half strength MS medium supplemented with 2.0 mgL^−1^ indole-3 butyric acid (IBA). All* in vitro* regenerated shoots were rooted by* ex vitro* method, and this has achieved 6-7 roots per shoot by pulsing of cut ends of the shoots using 200 as well as 300 mgL^−1^ IBA. The plantlets were hardened in the greenhouse for 4-5 weeks. The hardened plantlets were shifted to manure containing nursery polybags after five weeks and then transferred to a sand bed for another four weeks for acclimatization before field planting with 88% survival rate.

## 1. Introduction

*Passiflora edulis* Sims f.* flavicarpa* Deg. (passion fruit) is an important species of the family Passifloraceae, distributed mainly in the tropical and the subtropical regions of the world. It is native to Brazil and the fruits are mainly used for processing of juice. The fruits are famous for aromatic flavor and rich nutritional and medicinal properties. These are well known for their delicious juice, considered to be an instant energy drink in many parts of the world, particularly in South America, Australia, New Zealand, and South Africa [1].

Passion fruit vines are found wild and cultivated also to some extent in many parts of the world. In Brazil, it is cultivated at commercial scale and the fruits are consumed as juices and in ice cream making [2]. The cultivation of passion fruit has also been taken up at commercial scale in North-East and South India to produce value-added products and to generate extra income for the farmers. It can be grown as intercrop during any seasons. Flowers are hermaphrodites and are violet or blue to pale violet colored, in axillary solitary cymes [3].

*P. edulis* yields essential oils used in perfumery and soap industry, and the products derived from this plant are internationally recognized as herbal medicines [4]. This species is used in several pharmaceutical preparations in Brazil. The Italian chemists have extracted passiflorine from the air-dried leaves of* P. edulis.* The fruits contain vital antioxidants found to inhibit the growth of cancer cells [3]. In Madeira, the juice of passion fruits is given as digestive stimulant and to treat gastric cancer [5].

Passion fruit is rich in saponins, alkaloids, tannins, flavonoids, vitamins, and free amino acids, namely, arginine, aspartic acid, glycine, leucine, lysine, proline, threonine, tyrosine, and valine. The seeds yield 23% oil which is similar to sunflower and soybean oil and have industrial uses. It is also known to possess antibacterial, antiseptic, astringent, antiulcer, anti-inflammatory, spermicidal, and anticancer properties [6, 7].

Passion fruit species are normally propagated through seeds and stem cuttings. The vegetative propagation method (through stem cuttings) is most popular all over the world to maintain all essential superior characters of the genotype like disease resistance, size of fruit, juice content, time of maturity, and so forth. But this vine is affected by several viral, bacterial, and fungal diseases which caused heavy loss to the growers [8]. The vegetative propagation method causes the carry-over of disease-causing microorganisms from mother plant to the next generation [9]. Efficient micropropagation protocol for* Passiflora* species and its hybrids may play an important role in the production of healthy and disease-free stock plant material which can be used as source of medicinal herbal products, nutritional fruits, and ornamental flowers [10].

Biotechnology methods with selection of shoot apical and nodal meristems as source of explants can be used for rapid multiplication for improved varieties and to produce disease free quality planting material [11]. Some earlier work is available on this medicinal plant species [10, 12–16]. The present study describes an efficient protocol in terms of number of shoots induced from each node of explants, number of shoots multiplied, success in* ex vitro* rooting, and higher rate of survival of plantlets under natural conditions after hardening in the greenhouse.

## 2. Materials and Methods

### 2.1. Source Plant and Explant Collection

Explanting material of* Passiflora edulis* Sims f.* flavicarpa* Deg. was collected from the Coromandel Coastal Region of South India (including Tamil Nadu and Puducherry) during the months of February to December, 2013. Healthy, soft, and juvenile branches were collected from a one-year-old vine and brought to the laboratory. The leaves were excised and the stem (nodal segments) was cut into segments (2-3 cm long), each with at least one node.

### 2.2. Pretreatment and Surface Sterilization

The explants were pretreated with 0.1% (w/v) Bavistin (a systemic fungicide; BASF India Ltd., India) solution, and subsequently the surface was sterilized with 0.1% (w/v) HgCl_2_ (disinfectant, Himedia, India) solution for 5 min to check fungal and bacterial contamination, respectively. After rinsing five to six times with sterile distilled water, the explants were dipped in 90% ethyl alcohol. The sterilized explants were inoculated vertically onto the culture medium under laminar air flow cabinet (Technico Pvt. Ltd., Chennai, India).

### 2.3. Culture Medium, Culture Conditions, and Initiation of Multiple Shoots

Murashige and Skoog [17] medium (MS) was used as basal medium in the present study which was supplemented with 3% (w/v) sucrose and 0.8% (w/v) agar (Himedia, India). MS medium augmented with BAP and Kin ranging from 1.0 to 5.0 mgL^−1^ was used for the initiation of the shoots from nodal meristem of the explants. The pH of the media was adjusted to 5.8 using either 0.1 N NaOH or 0.1 N HCl prior to autoclaving the medium. Ten mL of medium (10 replicates) was poured in each culture tube. All the experiments were repeated thrice. The cultures were incubated under a 12 h photoperiod in cool white fluorescent light (44-45 *μ*mol m^−2^ s^−1^ Spectral Flux Photon, SFP) intensity.

### 2.4. Multiplication of Shoots

The shoots regenerated* in vitro* from the meristem of nodal explants were used for further multiplication of the shoots. The cultures were multiplied by two approaches: (i) the mother explants were repetitively transferred to fresh medium for 2-3 passages after harvesting* in vitro* raised shoots and (ii) the* in vitro* produced shoots were cut into 2–4 cm long segments (each with at least 1-2 nodes) and subcultured on fresh medium. The MS medium supplemented with cytokinins (BAP and Kin) ranging from 0.5 to 2.5 mgL^−1^ was used for multiplication of shoots. About 100 mL of medium (10 replicates) was poured in each culture flask. All the experiments were repeated thrice. The cultures were maintained at 25 ± 2°C temperature and 40–45 *μ*mol m^−2^ s^−1^ SFP light under 12:12 hrs light: dark photoperiod. Regular subculturing was performed after every four to five weeks.

### 2.5. Induction of Roots from the Shoots

The elongated* in vitro* produced shoots (3–5 cm long) were excised from the 4-week-old cultures and used for rooting experiments. The excised shoots were transferred to 1/4th, half and full strength agar-gelled MS basal medium supplemented with different concentrations of IBA and *α*-Naphthalene acetic acid (NAA) ranging from 0.5 to 3.0 mgL^−1^ to induce roots* in vitro*. Ten mL of this medium with 10 replicates was poured in each culture tube for root induction from the cut end of the shoots. Culture conditions were the same as for shoot multiplication except for the intensity of light (diffused light of 15–20 *μ*mol m^−2^s^−1^ SFP).

### 2.6. *Ex Vitro* Root Induction from the* In Vitro* Raised Shoots

Experiments were conducted to achieve rooting and hardening simultaneously using* ex vitro* method to save energy, cost of production, and time. The shoots were treated with IBA and NAA (50 to 500 mgL^−1^) solutions for five min and transferred to the ecofriendly plain paper cups (size 150 mL; Vandana Paper Products, Chennai, India) containing 55 g autoclaved soilrite (a mixture of perlite, Irish Peat Moss, and exfoliated vermiculite; KelPerlite, Bangalore, India), moistened with 10 mL aqueous 1/4th MS salts solution by the interval of one week and maintained in the greenhouse for five weeks. The experimental cups were kept in the greenhouse for root induction as well as hardening of the plantlets simultaneously.

### 2.7. Hardening and Acclimatization of Plantlets

After one month, the* in vitro* rooted shoots were taken out from the medium and washed with autoclaved distilled water to remove all traces of medium and agar gel. These individual plantlets were transferred to paper cups containing soilrite which was covered with transparent plastic cups (size 200 mL; Swastik PolyPack, Chennai, India) in inverted position. These setups were placed in the greenhouse for acclimatization and hardening. After optimizing the growth of the rooted plantlets, these were transferred to nursery polybags containing garden soil, organic matter, soilrite, and sand (1 : 1 : 1 : 1).

### 2.8. Statistical Data Analyses

The experiments were completely carried out with 10 replicates and repeated thrice. Data were subjected to analysis of variance by ANOVA and the significance of differences was calculated by Duncan's Multiple Range Test using SPSS software (version 16.0).

## 3. Results and Discussion

### 3.1. Establishment of Cultures

**Figure 1:**
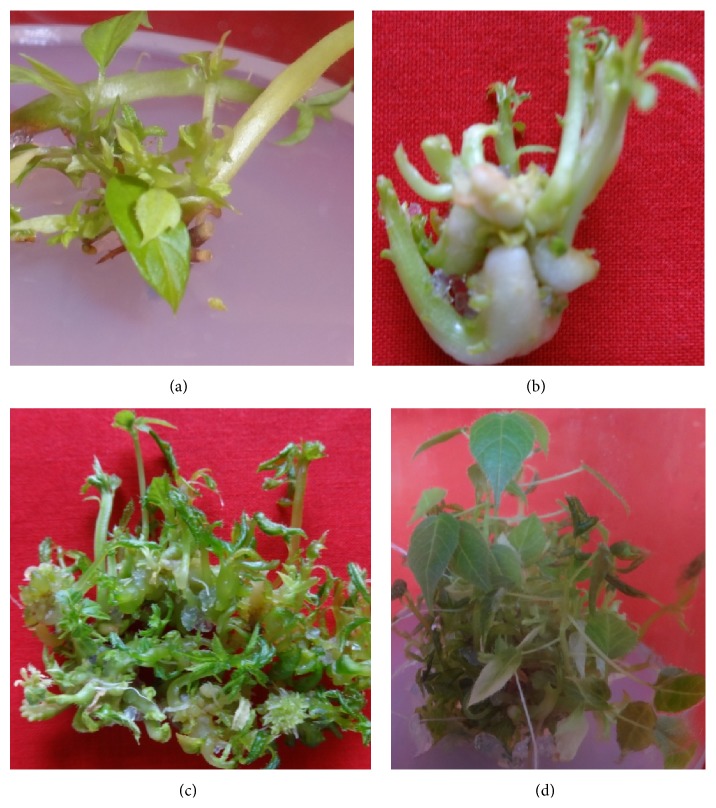
(a) Initiation of shoots from the nodal meristem. (b) Multiple shoots from the explants on MS medium with BAP. (c) Multiplication of shoots after two weeks. (d) Multiplication of shoots after four to five weeks.

Shoot bud initiation from nodal meristems of explants occurred after five-six days of inoculation. Fresh but thick shoot segments were found most suitable for culture initiation. All the nodal segments (100%) were sterilized with 0.1% HgCl_2_ solution. It was difficult to sterilized mature explants which were collected during the months of April to June and took more time (4-5 weeks) to initiate the shoot buds from the nodal meristems in cultures. Numerous shoots (8.21 ± 1.13 shoots per explant) with 2-3 cm length were reported on MS medium supplemented with 2.0 mgL^−1^ BAP (Figures 1(a) and 1(b)). A less number of shoots (3-4 shoots per explant) were differentiated on MS medium augmented with Kin (Table 1). Among the cytokinins, BAP was reported to be the most appropriate for initiation of cultures with MS medium. The rejuvenation of meristem was achieved through selection of explants and by treatment of different cytokinins. Ragavendran et al. [16] used node and shoot tip explants of* P. foetida* and regenerated 1-2 shoots per explant on MS medium supplemented with BAP.

*In vitro* propagation by nodal cuttings promoted the development of a preexistent morphological structure, and the nutritional and hormonal conditions of the medium break the dormancy of the axillary bud which promoted its rapid development [18]. Organogenesis in passion fruit has also been reported by some researchers [19, 20], culturing different types of explants in media supplemented with BAP.* In vitro* multiplication of* Passiflora edulis* by direct organogenesis through nodal cuttings was based on the concept that the higher the number of nodes the higher the number of plantlets. Trevisan and Mendes [15] studied the development of adventitious buds from the leaf discs on BAP or Thidiazuron (TDZ) and reported 5.6 shoots on BAP + coconut water containing medium. Effectiveness of BAP over Kin for shoot initiation from the buds has been reported in a number of other plant species like* Ceropegia bulbosa* [21],* Momordica dioica* [22],* Leptadenia reticulata* [23], and* Turnera ulmifolia* [24].

### 3.2. Multiplication of Shoots* In Vitro*

The shoots were multiplied by repeated transfer of mother explants of* P. edulis* on MS medium fortified with 1.0 mgL^−1^ of each BAP and Kin. This process of shoot amplification has been adopted by many researchers [21, 25, 26]. On adopting this process, 25.73 ± 0.06 shoots per vessel were produced after 2-3 passages (Table 2). This media composition was found good for shoot elongation also. Dornelas and Vieira [19] multiplied* P. edulis* shoots on MS medium supplemented with BAP or BAP + NAA. Hall et al. [20] used BAP + coconut water to culture and multiply the shoots of passion fruit. However, TDZ has also been reported as effective growth regulator for adventitious shoot multiplication in several crop plants [27–29]. Drew [12] cultured axillary buds of different* Passiflora* species on MS medium supplemented with BAP, 2iP (N6-iso pentenyl adenine), or IAA and developed some shoots. The protocol reported here improved the number of shoots multiplied* in vitro* per explant and thus shows higher efficiency than previously employed methods.

The shoots and leaves of the* in vitro* multiplied shoots were small in the first and second weeks of the incubation (Figure 1(c)) but the size of leaves was enlarged and the shoots were elongated in the last two weeks (Figure 1(d)). Well-developed leaf-system supports the chances of survival of* in vitro* raised plantlets during hardening and field transfer [30]. Plantlets with a high number of well-developed leaves are more efficient photosynthetically and therefore adapt quickly to natural environment as compared to those with smaller and fewer leaves [31].

After the establishment of cultures* in vitro*, some of the MS medium contents (e.g., sucrose) were replaced by cheaper materials available in the local market. This could be achieved through the use of locally available, cost effective alternatives like sugar cubes and sugar crystals in the place of sucrose [22]. It was reported that the number of shoots multiplied were remain more or less same with the alternate source of carbon in present study.

### 3.3. *In Vitro* Rooting of Microshoots

**Figure 2:**
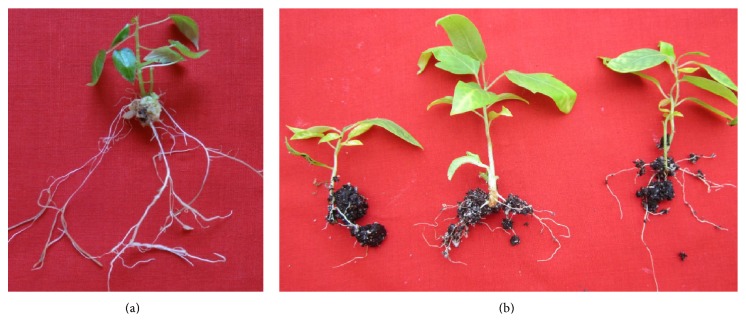
(a)* In vitro* root induction from the shoots on half strength MS medium with IBA. (b)* Ex vitro* root formation in the greenhouse after four weeks.

Roots have an essential role in plant growth and development in supplying water and nutrients to the plant from the environment [32]. About 98% of the shoots were rooted on 1/2 strength of MS medium supplemented with IBA. IBA was reported most effective in induction of roots from the cut ends of the shoots in present study. About 63% and 82% of the shoots, with less number of roots, were rooted on full and 1/4 strength MS medium, respectively (Table 3). Callus formation (moderate) was also observed when the shoots were rooted with full strength MS medium supplemented with 2.0 mgL^−1^ IBA. Maximum number of shoots was reported on half strength MS medium supplemented with 2.0 mgL^−1^ IBA (Figure 2(a)). Each shoot produced 7.13 ± 0.56 roots within 3-4 weeks on this medium combination (Table 4). The highest percentage of shoots (73%) was rooted on NAA with less number of roots (5.8). Our result signifies that half strength of MS salts in medium is appropriate for* in vitro* rooting and is in line with the research work published by many authors [33–35]. Ragavendran et al. [16] also rooted* in vitro* raised shoots by use of IBA in case of* P. foetida*.

### 3.4. *Ex Vitro* Root Induction

We reported 100% rooting response when the excised shoots were pulse treated with IBA solutions for* ex vitro* rooting experiments. This is the first report on the* ex vitro* rooting of shoots of* P. edulis*. Maximum response and number and length of roots were reported with IBA at 200 mgL^−1^ concentration and almost the same number of roots per shoot was observed when the shoots were treated with 300 mgL^−1^ IBA. Less number of roots (maximum 6.3 roots per shoot) was recorded with NAA concentrations (Table 5). Maximum number of roots (6.70 ± 0.37) was reported with 200 mgL^−1^ IBA in this study (Figure 2(b)). It is a cost effective technique and could save time and energy in plant propagation system [36–38].* Ex vitro* root induction was successfully proved by many researchers in* Ceropegia bulbosa* [21],* Leptadenia reticulata* [23],* Caralluma edulis* [33], and so forth. It has been reported that* ex vitro* rooted plantlets are better suited to tolerate environmental stresses [39, 40].

### 3.5. Hardening and Acclimatization of Plantlets

**Figure 3:**
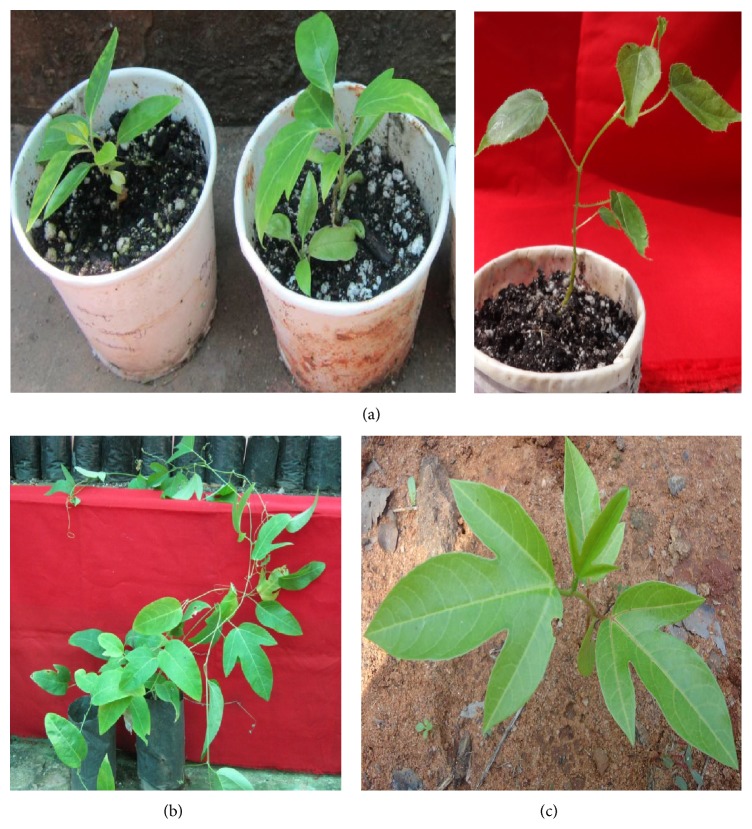
(a) Hardening of plantlets in the greenhouse. (b) Plantlets in nursery polybags. (c) Acclimatized plant of passion fruit growing in the natural conditions.

The* in vitro* as well as* ex vitro* rooted plantlets were hardened in the greenhouse. After 30–35 days, rooting was recorded in* ex vitro* rooted shoots. Transparent polythene cup caps were gradually loosened and finally removed after 30 days (Figure 3(a)). Plants were then transferred to nursery polybags for another 4-5 weeks (Figure 3(b)). About 88% of the plants were hardened successfully. Hardened and acclimatized plants were shifted to the soil beds (Figure 3(c)). The acclimatized plants exhibited normal growth and true-to-type morphology under natural conditions.

### 3.6. Conclusion

The rate of shoot multiplication was very high in the present report. The good success rate has been achieved in* ex vitro* rooting which saved time, energy, and cost of production of micropropagated plantlets. The developed method offers an alternative for mass propagation of disease-free stock plant material of* Passiflora edulis*. This could greatly enhance availability of superior and healthy passion fruit planting materials at an affordable cost to the farmers.

## Figures and Tables

**Table 1:** Effect of cytokinins (BAP and Kin) on induction of shoots from explants of *P. edulis* after 4 weeks.

Conc. of BAP (mgL^−1^)	Conc. of Kin. (mgL^−1^)	Number of shoots/explant (mean ± SD)	Response (%)
Control (0.0)	(0.0)	0.00 ± 0.00	(0.0)
1.0	—	6.02 ± 0.37^d^	93
2.0	—	8.21 ± 1.13^f^	100
3.0	—	7.23 ± 0.71^e^	100
4.0	—	7.56 ± 0.56^e^	99
5.0	—	6.19 ± 0.45^d^	91
—	1.0	2.51 ± 0.33^a^	73
—	2.0	3.20 ± 0.57^bc^	87
—	3.0	2.73 ± 1.68^ab^	85
—	4.0	3.19 ± 0.83^bc^	79
—	5.0	3.74 ± 0.23^c^	64

The Experiments were carried out with 10 replicates and repeated thrice. Mean separation was analyzed by ANOVA using SPSS software (var. 16.0) and significance variation between the concentrations was studied using DMRT at 0.05% level. Superscript letters denote the highest/lowest significant value within the concentrations/groups in this study. The same superscript letters are not significantly different according to DMRT at *P* < 0.05.

**Table 2:** Effect of cytokinins (BAP and Kin) after 4 weeks on multiplication of shoots.

Conc. of BAP (mgL^−1^)	Conc. of Kin. (mgL^−1^)	Number of shoots (mean ± SD)	Length of shoots (cm) (mean ± SD)
Control (0.0)	(0.0)	0.00 ± 0.00	0.00 ± 0.00
0.5	0.5	19.55 ± 0.03^c^	4.67 ± 0.43^c^
1.0	1.0	25.73 ± 0.06^e^	5.33 ± 0.06^d^
1.5	1.5	22.47 ± 0.41^d^	4.81 ± 0.22^cd^
2.0	2.0	17.76 ± 0.66^b^	3.45 ± 0.19^b^
2.5	2.5	16.34 ± 0.54^a^	2.26 ± 0.33^a^

The Experiments were carried out with 10 replicates and repeated thrice. Mean separation was analyzed by ANOVA using SPSS software (var. 16.0) and significance variation between the concentrations was studied using DMRT at 0.05% level. Superscript letters denote the highest/lowest significant value within the concentrations/groups in this study. The same superscript letters are not significantly different according to DMRT at *P* < 0.05.

**Table 3:** Effect of strength of MS medium augmented with 2.0 mgL^−1^ IBA on *in vitro* root initiation from shoots of *P. edulis* after 4 weeks.

Strength of MS medium	Response (%)	Number of roots (mean ± SD)	Intensity of callus
Full strength	63	4.30 ± 0.15^a^	Moderate callus
Half strength	98	7.13 ± 0.56^c^	No callus
1/4 strength	82	6.46 ± 0.22^b^	No callus

The Experiments were carried out with 10 replicates and repeated thrice. Mean separation was analyzed by ANOVA using SPSS software (var. 16.0) and significance variation between the concentrations was studied using DMRT at 0.05% level. Superscript letters denote the highest/lowest significant value within the concentrations/groups in this study. The same superscript letters are not significantly different according to DMRT at *P* < 0.05.

**Table 4:** Effect of auxins (IBA, NAA) on *in vitro* root induction from *in vitro* raised shoots after 4 weeks.

Conc. of IBA (mgL^−1^)	Conc. of NAA (mgL^−1^)	Number of roots (mean ± SD)	Response (%)
Control (0.0)	(0.0)	0.00 ± 0.00	0
0.5	—	1.80 ± 0.03^b^	53
1.0	—	3.64 ± 0.74^c^	77
1.5	—	5.81 ± 0.45^e^	92
2.0	—	7.13 ± 0.56^f^	98
2.5	—	6.67 ± 0.07^f^	97
3.0	—	6.54 ± 0.35^f^	97
—	0.5	1.22 ± 0.35^a^	34
—	1.0	3.16 ± 0.04^c^	56
—	1.5	4.83 ± 0.27^d^	69
—	2.0	5.48 ± 0.43^e^	73
—	2.5	5.87 ± 0.91^e^	73
—	3.0	5.61 ± 0.22^e^	71

The Experiments were carried out with 10 replicates and repeated thrice. Mean separation was analyzed by ANOVA using SPSS software (var. 16.0) and significance variation between the concentrations was studied using DMRT at 0.05% level. Superscript letters denote the highest/lowest significant value within the concentrations/groups in this study. The same superscript letters are not significantly different according to DMRT at *P* < 0.05.

**Table 5:** Effect of auxins (IBA, NAA) on *ex vitro* roots induction in the greenhouse after 5 weeks.

Conc. of IBA (mgL^−1^)	Conc. of NAA (mgL^−1^)	Number of roots (mean ± SD)	Response (%)
Control (0.0)	(0.0)	0.00 ± 0.00	0
50	—	3.15 ± 0.04^b^	81
100	—	4.37 ± 0.02^c^	93
200	—	6.70 ± 0.37^fg^	100
300	—	6.56 ± 0.23^fg^	100
400	—	6.11 ± 0.71^efg^	100
500	—	6.04 ± 0.02^ef^	100
—	50	2.15 ± 0.09^a^	76
—	100	4.32 ± 0.02^c^	82
—	200	6.28 ± 0.34^efg^	88
—	300	6.34 ± 0.61^efg^	93
—	400	5.76 ± 0.38^e^	96
—	500	5.20 ± 0.02^d^	98

The Experiments were carried out with 10 replicates and repeated thrice. Mean separation was analyzed by ANOVA using SPSS software (var. 16.0) and significance variation between the concentrations was studied using DMRT at 0.05% level. Superscript letters denote the highest/lowest significant value within the concentrations/groups in this study. The same superscript letters are not significantly different according to DMRT at *P* < 0.05.

## Abbreviations

BAP: 6-Benzylaminopurine
Kin: Kinetin
IBA: Indole-3-butyric acid
NAA: *α*-Naphthalene acetic acid
MS medium: Murashige and Skoog (1962) medium
SFP: Spectral Flux Photon.
